# The Integrative Conjugative Element (ICE) of *Mycoplasma agalactiae*: Key Elements Involved in Horizontal Dissemination and Influence of Coresident ICEs

**DOI:** 10.1128/mBio.00873-18

**Published:** 2018-07-03

**Authors:** Eric Baranowski, Emilie Dordet-Frisoni, Eveline Sagné, Marie-Claude Hygonenq, Gabriela Pretre, Stéphane Claverol, Laura Fernandez, Laurent Xavier Nouvel, Christine Citti

**Affiliations:** a IHAP, Université de Toulouse, INRA, ENVT, Toulouse, France; b Centre de Génomique Fonctionnelle, Pôle Protéomique, Université de Bordeaux, Bordeaux, France; Northern Arizona University

**Keywords:** evolution, functional genomics, horizontal gene transfer, integrative conjugative element, *Mycoplasma*

## Abstract

The discovery of integrative conjugative elements (ICEs) in wall-less mycoplasmas and the demonstration of their role in massive gene flows within and across species have shed new light on the evolution of these minimal bacteria. Of these, the ICE of the ruminant pathogen Mycoplasma agalactiae (ICEA) represents a prototype and belongs to a new clade of the Mutator-like superfamily that has no preferential insertion site and often occurs as multiple chromosomal copies. Here, functional genomics and mating experiments were combined to address ICEA functions and define the minimal ICEA chassis conferring conjugative properties to M. agalactiae. Data further indicated a complex interaction among coresident ICEAs, since the minimal ICEA structure was influenced by the occurrence of additional ICEA copies that can *trans*-complement conjugation-deficient ICEAs. However, this cooperative behavior was limited to the CDS14 surface lipoprotein, which is constitutively expressed by coresident ICEAs, and did not extend to other ICEA proteins, including the *cis*-acting DDE recombinase and components of the mating channel whose expression was detected only sporadically. Remarkably, conjugation-deficient mutants containing a single ICEA copy knocked out in *cds14* can be complemented by neighboring cells expressing CDS14. This result, together with those revealing the conservation of CDS14 functions in closely related species, may suggest a way for mycoplasma ICEs to extend their interaction outside their chromosomal environment. Overall, this report provides a first model of conjugative transfer in mycoplasmas and offers valuable insights into understanding horizontal gene transfer in this highly adaptive and diverse group of minimal bacteria.

## IMPORTANCE

Integrative conjugative elements (ICEs) are self-transmissible mobile genetic elements that are key mediators of horizontal gene flow in bacteria. Recently, a new category of ICEs was identified that confer conjugative properties to mycoplasmas, a highly adaptive and diverse group of wall-less bacteria with reduced genomes. Unlike classical ICEs, these mobile elements have no preferential insertion specificity, and multiple mycoplasma ICE copies can be found randomly integrated into the host chromosome. Here, the prototype ICE of Mycoplasma agalactiae was used to define the minimal conjugative machinery and to propose the first model of ICE transfer in mycoplasmas. This model unveils the complex interactions taking place among coresident ICEs and suggests a way for these elements to extend their influence outside their chromosomal environment. These data pave the way for future studies aiming at deciphering chromosomal transfer, an unconventional mechanism of DNA swapping that has been recently associated with mycoplasma ICEs.

## INTRODUCTION

Integrative conjugative elements (ICEs) are self-transmissible mobile genetic elements that are key mediators of horizontal gene flow in bacteria (1). These self-transmissible elements encode their excision and transfer by conjugation and integration into the genome of the recipient cell, where they replicate as a part of the host chromosome. Recently, a new family of self-transmissible integrative elements was identified in the genome of several mycoplasma species and confers conjugative properties to this important group of bacteria (2–8).

Mycoplasmas are well known for having some of the smallest genomes thus far characterized in free-living organisms, with many species being successful human and animal pathogens (9, 10). Mycoplasmas belong to the class *Mollicutes*, a large group of atypical bacteria that have evolved from low-GC, Gram-positive common ancestors (11). For decades, their evolution has been considered to be marked by a degenerative process, with successive losses of genetic material resulting in current mycoplasmas having no cell wall and limited metabolic capacities (9). The recent discovery of massive horizontal gene transfer (HGT) in mycoplasmas has shed new light on the dynamics of their reduced genomes (12, 13). Evidence for HGT in these minimal bacteria came from the identification of putative ICEs in several species together with *in silico* data suggesting that mycoplasma species of distant phylogenetic groups have exchanged a significant amount of chromosomal DNA (14).

**FIG 1:**
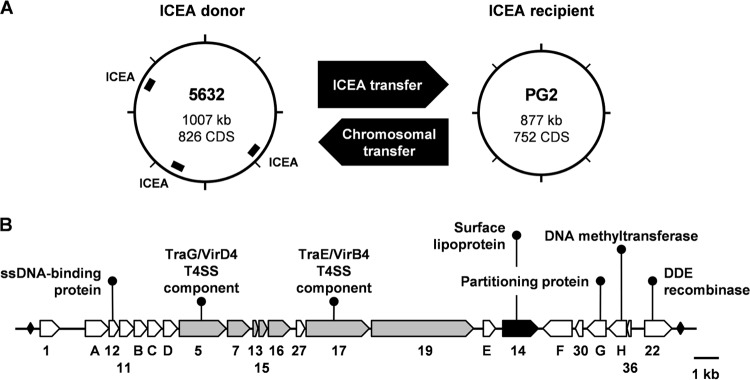
ICEA-mediated horizontal gene transfer (HGT) in M. agalactiae. (A) Schematic illustrating the two mechanisms of gene exchanges occurring upon mating experiments involving strain 5632 as ICE donor and strain PG2 as ICE recipient cells (7, 12). One of the three chromosomal ICEA copies of 5632 is transferred to PG2 and integrates randomly in the recipient genome (ICEA transfer). ICEA self-dissemination is associated with a second mechanism of gene exchange that occurs in the opposite direction from the recipient to the donor cells and involves large chromosomal DNA movements (chromosomal transfer). ICEA transfer confers conjugative properties to the PG2 recipient cells (7). (B) The 23 genes identified in ICEA are represented with their respective orientations and approximate nucleotide sizes. The two inverted repeats (IRs) flanking the ICEA are represented by black diamonds. The genes encoding predicted surface lipoproteins and those encoding proteins with putative transmembrane domains are in black and gray, respectively. Hypothetical functions were deduced from putative conserved domains found in several ICEA products (Table S1). ssDNA, single-stranded DNA; T4SS, type IV secretion system.

Conjugative properties of mycoplasmas were further demonstrated using the ruminant pathogen Mycoplasma agalactiae as a model organism (7, 12). In this species, mating experiments and associated next-generation sequencing analyses established that mycoplasma ICEs (MICEs) are self-transmissible mobile elements conferring to recipient cells the capacity to conjugate (Fig. 1). These uncovered at the same time an unconventional conjugative mechanism of chromosomal transfers (CTs) which involved large chromosomal regions and were independent of their genomic locations (12). While ICE self-dissemination from ICE-positive to ICE-negative cells was documented, CTs were observed in the opposite direction, resulting in the incorporation of large genomic regions (Fig. 1). Remarkably, CTs can mobilize up to 10% of the mycoplasma genome in a single conjugative event generating complex progeny of chimeric genomes that may resemble conjugative distributive transfers in Mycobacterium smegmatis (15). While ICE and CTs appeared to represent two independent events, CTs most likely rely on ICE factors for providing the conjugative pore.

10.1128/mBio.00873-18.4 TABLE S1  Relevant features of ICEA products. Download TABLE S1, DOCX file, 0.05 MB. Copyright © 2018 Baranowski et al. 2018 Baranowski et al. This content is distributed under the terms of the Creative Commons Attribution 4.0 International license.

Among the MICEs described so far, the ICE of M. agalactiae (ICEA) has been most extensively studied (3, 16). ICEA and MICEs in general belong to a new family of self-transmissible integrative elements that rely on a DDE transposase of the prokaryotic Mutator-like family for their mobility (7, 17). Mainly associated with small and simple transposons such as insertion sequences, DDE transposases are also encoded by some more complex mobile elements, such as streptococcal Tn*GBS* conjugative transposons (17). Unlike Tn*GBS* conjugative transposons, which have a preferential insertion location upstream of σA promoters, ICEA integration occurs randomly in the host chromosome, generating a diverse population of ICEA transconjugants (7, 17). This situation also contrasts with the more conventional ICEs, which encode site-specific tyrosine recombinases (1). ICEA occurrence varies among M. agalactiae strains, with strain 5632 containing three nearly identical ICEA copies and strain PG2 containing no ICE or a vestigial form (14, 16). Functional ICEAs are about 27 kb long and are composed of 23 genes (Fig. 1), most of which encode proteins of unknown function (see Table S1 in the supplemental material) with no homologue outside the *Mollicutes* (3). Among the few exceptions are CDS5 and CDS17, two proteins with similarity to conjugation-related TraG/VirD4 and TraE/VirB4, respectively (Table S1). Both proteins are energetic components of the type IV secretion systems, which are usually involved in DNA transport (18).

The establishment of laboratory conditions in which ICE transfer can be reproduced and analyzed in M. agalactiae (7), together with the development of specific genetic tools for the manipulation of this species (19), offers a unique opportunity to further investigate the detailed mechanisms underlying HGTs in mycoplasmas. In the present study, a transposon-based strategy was devised to knock out individual ICEA genes and to decipher ICEA functions in M. agalactiae. Data showed that the minimal ICEA chassis required for conferring conjugative properties to M. agalactiae was influenced by the occurrence of additional ICEA copies that can *trans*-complement conjugation-deficient ICEAs. Complementation studies further unveiled the complexity of this interplay, which can even extend to neighboring cells, and the key role played by the coresident ICEA expression pattern. This report is a first step toward understanding HGT in *Mollicutes* and provides a valuable experimental framework to decipher the mechanisms of DNA exchange in more-complex bacteria associated with this new category of mobile elements.

## RESULTS

### Conjugative properties of mutated Mycoplasma agalactiae ICEA.

**FIG 2:**
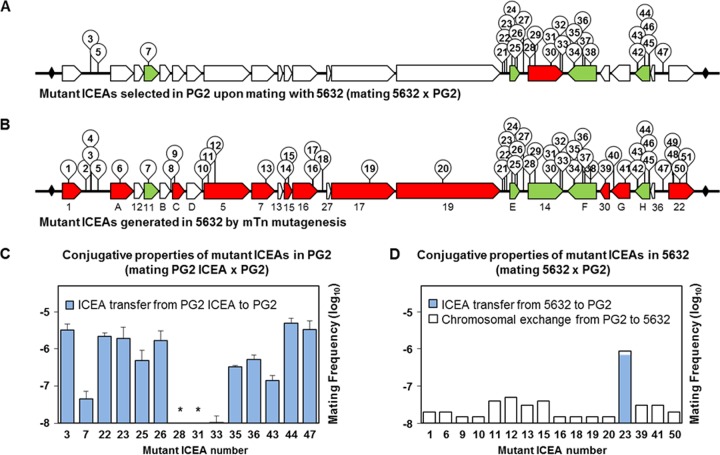
Functional analysis of mutant ICEAs in the 5632 and PG2 genetic backgrounds. (A and B) Schematic illustrating the 51 mutant ICEAs generated by transposon mutagenesis in M. agalactiae strain 5632 (B) and the mutant ICEAs selected in PG2 upon mating with 5632 (A). Individual mutant ICEAs are designated according to Table S2. The genes with no mTn insertion are indicated in white. ICEA genes found essential (red) or dispensable (green) were identified according to their genetic backgrounds, which differ with respect to ICEA content (Fig. 1). (C and D) Conjugative properties of selected mutant ICEAs in PG2 (C) and 5632 (D). Mating frequencies were calculated as the number of dually resistant transconjugants per total CFU (mating frequencies per singly resistant CFU are provided in Table S3). Donor cells were mated with a pool of 5 ICEA-negative PG2 clones encoding resistance to puromycin (mating PG2 ICEA × PG2) or tetracycline (mating 5632 × PG2). Dually resistant colonies were selected by using a combination of gentamicin and puromycin (mating PG2 ICEA × PG2) or of gentamicin and tetracycline (mating 5632 × PG2). For PG2 ICEA × PG2 matings (C), the data represent means of results from at least three independent assays, with the exception of mutant ICEA 23 (nine independent assays). Since mutant ICEAs can be found integrated at different genomic positions, two PG2 ICEA transconjugants were used for mutant ICEA 7 (ICEA at genomic positions 395291 and 433901). Standard deviations are indicated by error bars. The asterisk indicates a mating frequency below the detection limit (1 × 10^−10^ transconjugants per total CFU). For 5632 × PG2 matings (D), the data represent averages of results from two independent assays. The genetic profile of the transconjugants was determined using 10 to 166 dually resistant colonies per mating, which, for lower mating frequencies, represented nearly all the progeny.

To elucidate the molecular mechanisms underlying ICE conjugative transfer in M. agalactiae, a library of 1,440 individual mutants was generated by random insertion of a minitransposon (mTn) into the genome of strain 5632, which contains three nearly identical copies of a functional ICEA (Fig. 1). Mating experiments were conducted using pools of 96 individual 5632 mutants as donors and a pool of 5 PG2 recipient clones to avoid possible bias associated with a particular variant. Donors and recipients were chosen to carry compatible antibiotic markers (see Materials and Methods), and the resulting transconjugants were obtained with a frequency ranging from 2 × 10^−9^ to 8 × 10^−8^ transconjugants/total CFU, as expected for the 1:10 ratio (5632/PG2) that favors ICEA transfer from 5632 to PG2 (7). Doubly resistant colonies were further subjected to detailed genetic analysis to (i) identify ICEA-positive PG2 versus 5632 that had acquired PG2 genomic materials by CTs and (ii) map the mTn position within ICEA-positive PG2 transconjugants. This strategy allowed the identification of 27 unique mutant ICEAs (Fig. 2A; see also Table S2 in the supplemental material). Remarkably, mTn insertions were found to cluster within a 6.4-kb ICEA region spanning *cdsE* to *cdsH*, with the exception of three inserted in the noncoding regions *ncr1*/*A* and *ncr36*/*22* (Fig. 2A, mutants 3, 5, and 47) and one inserted in *cds11* (Fig. 2A, mutant 7).

10.1128/mBio.00873-18.5 TABLE S2  Mutant ICEAs generated in M. agalactiae strain 5632. Download TABLE S2, DOCX file, 0.1 MB. Copyright © 2018 Baranowski et al. 2018 Baranowski et al. This content is distributed under the terms of the Creative Commons Attribution 4.0 International license.

10.1128/mBio.00873-18.6 TABLE S3  Mating frequencies per singly resistant CFU. Download TABLE S3, DOCX file, 0.1 MB. Copyright © 2018 Baranowski et al. 2018 Baranowski et al. This content is distributed under the terms of the Creative Commons Attribution 4.0 International license.

Since PG2 transconjugants contain no coresident ICEA copies (see Fig. S1 in the supplemental material), mating experiments were performed to evaluate the conjugative properties of selected mutant ICEAs (Fig. 2A). Individual PG2 transconjugants (further designated PG2 ICEA) were mated with a pool of five ICEA-negative PG2 clones as recipient cells (Fig. 2C). The PG2 ICEA cells carrying an mTn inserted in *ncr1*/*A*, *ncr19*/*E*, and *ncr36*/*22* (Fig. 2A and C, mutants 3, 22, 23, and 47) displayed comparable mating frequencies (1.9 × 10^−6^ to 3.5 × 10^−6^ transconjugants/total CFU), suggesting that mTn insertions in these regions had no or minimal effect on conjugation. Conversely, mating experiments involving *cds14* knockout ICEAs in PG2 (Fig. 2A and C, mutants 28, 31, and 33) confirmed the essential role previously recognized for this gene (7) and further indicated that *cds14* can be complemented in *trans* by coresident ICEA copies, such as in 5632. The insertion of an mTn in *cds11*, *cdsE*, *cdsF*, and *cdsH* (Fig. 2A and C, mutants 7, 25 to 26, 35 to 36, and 43 to 44) did not abrogate ICEA transfer, but several mutant ICEAs displayed a reduced capacity to self-disseminate. Whether the conjugative properties of these PG2 ICEA cells can be influenced by the chromosomal position of the integrated ICEA is unknown. However, similar mating frequencies (4 × 10^−8^ to 5 × 10^−8^ transconjugants/total CFU) were observed for two PG2 transconjugants sharing the same mutant ICEA (Fig. 2C, mutant 7) integrated at different chromosomal sites (genomic positions 395291 and 433901). These results identified *cdsE*, *cdsF*, *cdsH*, and, to a lesser extent, *cds11* as dispensable for ICEA self-dissemination.

10.1128/mBio.00873-18.1 FIG S1  PG2 ICEA cells harbor a single ICEA copy randomly integrated into the host chromosome. Southern blotting experiments (E. Dordet-Frisoni, M. S. Marenda, E. Sagné, L. X. Nouvel, R. Guérillot, P. Glaser, A. Blanchard, F. Tardy, P. Sirand-Pugnet, E. Baranowski, and C. Citti, Mol Microbiol 89:1226–1239, 2013, doi:10.1111/mmi.12341) performed with PG2 ICEA transconjugants failed to reveal multiple ICEA integrations. (A and B) Mycoplasma genomic DNAs were restricted with EcoRV and hybridized with mTn gentamicin (Gm)-specific (A) or ICEA *cds22*-specific (B) probes. The identification of a single Gm-positive DNA fragment in PG2 ICEA transconjugants was in agreement with genomic DNA sequencing data indicating a single mutant ICEA insertion in the PG2 chromosome. This result was further supported by the results of DNA hybridization with the *cds22*-specific probe that also discarded any wild-type ICEA copy in PG2 ICEA transconjugants. Differences in size between *cds22-*positive DNA fragments are consistent with the random insertion of ICEA in the host chromosome. The digested ICEA circular form is indicated by an arrow. (C) For each of the PG2 ICEA transconjugants, the size of the Gm-positive DNA fragments was in agreement with predicted values. The number in parenthesis indicates the size of the ICEA fragment with an inserted mTn. The Gm- and *cds22-*positive fragments are indicated by asterisks (*, *Gm*-specific probe; **, *cds22*-specific probe). Dashed lines indicate a fragment overlapping ICEA and genomic DNA. Download FIG S1, TIF file, 2.1 MB. Copyright © 2018 Baranowski et al. 2018 Baranowski et al. This content is distributed under the terms of the Creative Commons Attribution 4.0 International license.

### The minimal ICE chassis that confers conjugative properties to Mycoplasma agalactiae.

As shown above, mutant ICEs recovered in PG2 transconjugants displayed a biased distribution of their mTn insertions (Fig. 2A). This raised the issue of the representativeness of the 5632 library, and thus a PCR-based screening strategy for the direct identification of mutant ICEAs in 5632 was developed: mTn insertions across the entire ICEA were searched by a series of PCR assays using one primer matching each end of the mTn and one specific-ICEA primer selected from a set of oligonucleotides spanning the whole ICEA region. Amplifications were performed using pools of 96 individual mutants until one mTn insertion event per gene was detected, and positive pools were further characterized down to the single-mutant level. For each mutant, the mTn insertion was mapped by genomic DNA sequencing, which also confirmed the presence of a single mTn per chromosome. Finally, the distribution of mTn insertions among the three ICEA copies of 5632 was determined by long-range PCR amplifications using mTn-specific primers and a panel of oligonucleotides that are complementary to genomic DNA regions surrounding each ICEA copy. This strategy led us to identify 35 unique mutants (Table S2) among the three ICEA copies of 5632 (ICEA-I [29%], ICEA-II [37%], and ICEA-III [34%]). This time, mTn insertions were found broadly distributed throughout the ICEA locus with the exception of several genes (*cds12*, *cdsB*, *cdsD*, *cds13*, *cds27*, and *cds36*), all characterized by small sizes ranging from 0.20 to 0.65 kb. These findings indicate that the particular set of mutant ICEAs selected in PG2 as described above cannot be explained simply by the poor representativeness of the 5632 mutant library.

The 51 mutant ICEAs identified in 5632, either by PCR screening or by mating experiments, are illustrated in Fig. 2B. Of 35 mutant ICEAs identified by PCR, 24 did not correspond to detectable PG2 transconjugants previously obtained (compare Fig. 2A and B), suggesting that these mutant ICEAs have lost their capacity to disseminate from 5632 to PG2. This was confirmed by mating performed using each of these 5632 mutants individually as an ICEA donor (Fig. 2D) and by further analyses of their progeny. Results showed that when transconjugants were obtained, all displayed the 5632 genomic backbone of the mutant and corresponded to 5632 having acquired the second PG2 antibiotic marker upon CT. This was true for all except 5632 mutant 23 (mTn inserted in *ncr19*/*E* with no influence on conjugation), which was used as a positive control for ICEA transfer and which generated up to 97% PG2 transconjugants (Fig. 2D, mutant 23).

Overall, these results indicate that ICEA transfer can be abrogated or strongly affected by disrupting the genes encoding CDS1, CDSA, CDSC, CDS5, CDS7, CDS15, CDS16, CDS17, CDS19, CDS30, CDSG, and CDS22 in 5632 (Fig. 2B). Unlike the results seen with *cds14* knockout ICEAs, the conjugative properties of these mutant ICEAs cannot be restored by coresident ICEA copies. Finally, ICEA transfer was also abrogated by mTn insertion in *ncrD*/*5* and *ncr16*/*27* (Fig. 2B, mutants 10 and 18), raising questions about the presence of regulatory and/or *cis*-acting elements (e.g., *oriT*) in these regions. Whether short genes (<0.65 kb) with no mTn insertion may encode essential functions remains to be further investigated.

### ICEA transfer in Mycoplasma agalactiae requires the CDS14 surface lipoprotein.

**FIG 3:**
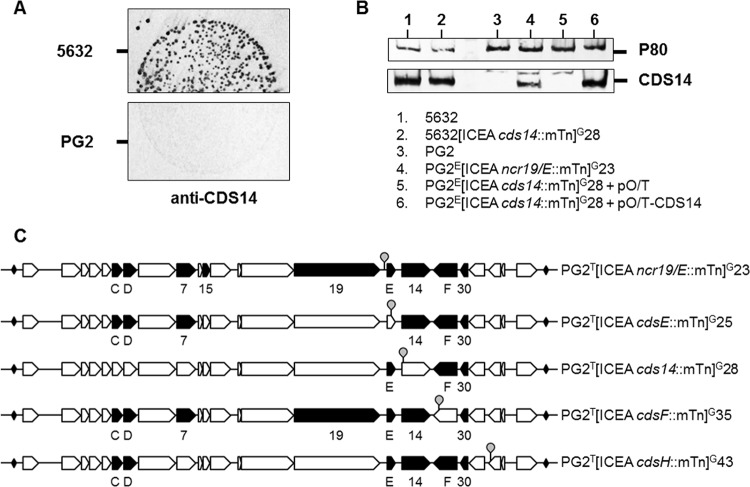
Protein expression of PG2-ICEA mutants. (A) Immunostaining of M. agalactiae colonies showing CDS14 lipoprotein expression at the surface of 5632 cells. Colony blotting was carried out by using a specific serum (anti-CDS14), and ICEA-negative PG2 cells (PG2) were used as a negative control. (B) Western blot analysis of CDS14 lipoprotein expression in 5632 and PG2 ICEA cells. CDS14 lipoprotein expression in strain 5632 containing three chromosomal ICEA copies (lane 1) was not abrogated in a 5632 mutant harboring a *cds14* knockout ICEA copy (lane 2). CDS14 lipoprotein expression was detectable in PG2 transconjugants that had acquired a mutant ICEA harboring an mTn inserted in *ncr19*/*E* (lane 4) but not in strain PG2 (lane 3) or in PG2 transconjugants harboring a *cds14* knockout ICEA (lane 5). Transformation of PG2 transconjugants harboring a *cds14* knockout ICEA with a plasmid expressing CDS14 restored the expression of the lipoprotein (lane 6). A specific serum raised against lipoprotein P80 was used as a control (P80). (C) Schematic illustrating the protein expression profiles of selected mutant ICEAs in PG2 cells. Mutant ICEAs are identified according to Table S2, and ICEA products detected by proteomics (Table S4) are indicated (closed arrows).

The CDS14 lipoprotein is essential for mycoplasma conjugation and contains a 27-amino-acid (aa) signal sequence (Fig. S2) that is characteristic of surface-exposed lipoproteins in M. agalactiae. The CDS14 surface location was confirmed in ICEA-positive cells by colony blotting assays using a specific antiserum (Fig. 3A), and the results are in agreement with proteomic data showing an association of CDS14 with the Triton X-114 hydrophobic fraction obtained after mycoplasma partitioning (16). Western blot analyses of 5632[ICEA *cds14*::mTn]^G^28 (mutant 28 in Fig. 2B; see also Table S2), which has one of the three ICEA copies with a knockout *cds14*, further demonstrated that this lipoprotein can be expressed by coresident ICEAs (Fig. 3B). This explains the capacity of *cds14* knockout ICEAs to be horizontally transferred from the 5632 cells as described above.

10.1128/mBio.00873-18.2 FIG S2  Global alignment of CDS14 lipoproteins found in ICEs of M. agalactiae strain 5632 (5632) and M. bovis strain PG45 (PG45). The alignments of CDS14 lipoprotein sequences derived from 5632 (MAGa5010) and PG45 (MBOVPG45_0187) were performed by using Needleman-Wunsch global alignment. The 27-aa sequence characteristic of surface-exposed lipoproteins is underlined. Download FIG S2, TIF file, 2.8 MB. Copyright © 2018 Baranowski et al. 2018 Baranowski et al. This content is distributed under the terms of the Creative Commons Attribution 4.0 International license.

10.1128/mBio.00873-18.7 TABLE S4  Proteomic analysis of PG2 ICEA mutants. Download TABLE S4, DOCX file, 0.05 MB. Copyright © 2018 Baranowski et al. 2018 Baranowski et al. This content is distributed under the terms of the Creative Commons Attribution 4.0 International license.

The role of the CDS14 lipoprotein was further investigated by using conjugation-deficient PG2^E^[ICEA *cds14*::mTn]^G^28, which has only one ICEA copy with an mTn inserted in *cds14*. Complementation studies confirmed that the conjugative properties of this mutant can be restored upon transformation with plasmid pO/T-CDS14, which expresses the wild-type CDS14, but not with the empty vector (Table 1, matings A and B). Remarkably, transformation of ICE-negative recipient cells with pO/T-CDS14 also restored the conjugative properties of PG2^E^[ICEA *cds14*::mTn]^G^28 (Table 1, mating C) with only a (ca. 20-fold) reduction in mating frequency. This result provides the first evidence of ICE complementation by neighboring cells and suggests that CDS14 lipoprotein may initiate ICEA transfer in M. agalactiae by promoting a contact between the donor cells and the recipient cells.

**TABLE 1:** Complementation studies with *cds14*, *cds5*, and *cds22* knockout ICEAs

Mating^a^	ICE donor^b^	ICE recipient^c^	Mating frequency (×10^−8^)^d^	Genomic profile of the mating progeny^e^	
				PG2	5632
Complementation of *cds14* knockout ICEAs					
A	PG2^E^[ICEA *cds14*::mTn]^G^28 + pO/T-CDS14	PG2^P^ + pO/T	170 ± 80	NA	NA
B	PG2^E^[ICEA *cds14*::mTn]^G^28 + pO/T	PG2^P^ + pO/T	0^f^	NA	NA
C	PG2^E^[ICEA *cds14*::mTn]^G^28 + pO/T	PG2^P^ + pO/T-CDS14	8.4 ± 6.3	NA	NA
D	PG2^E^[ICEA *cds14*::mTn]^G^28 + pO/T-CDS14bov^g^	PG2^P^ + pO/T	89 [33–145]	NA	NA
					
Complementation of *cds5* knockout ICEAs					
E	5632[ICEA *cds5*::mTn]^G^11 + pO/T-CDS5	PG2^P^ + pO/T	1 [0.5–2]	7	2
F	5632[ICEA *cds5*::mTn]^G^11 + pO/T	PG2^P^ + pO/T	0	NA	NA
G	5632[ICEA *cds5*::mTn]^G^12 + pO/T-CDS5	PG2^P^ + pO/T	1 [0.5–2]	14	8
H	5632[ICEA *cds5*::mTn]^G^12 + pO/T	PG2^P^ + pO/T	0	NA	NA
					
Expression of CDS5 truncated products					
I	5632[ICEA *ncr19*/*E*::mTn]^G^23 + pO/T-CDS5N1	PG2^P^ + pO/T	110 [96–124]	ND	ND
J	5632[ICEA *ncr19*/*E*::mTn]^G^23 + pO/T-CDS5C1	PG2^P^ + pO/T	210 [116–304]	ND	ND
K	5632[ICEA *ncr19*/*E*::mTn]^G^23 + pO/T-CDS5N2	PG2^P^ + pO/T	130 [112–148]	ND	ND
L	5632[ICEA *ncr19*/*E*::mTn]^G^23 + pO/T-CDS5C2	PG2^P^ + pO/T	180 [41–319]	ND	ND
M	5632[ICEA *ncr19*/*E*::mTn]^G^23 + pO/T	PG2^P^ + pO/T	110 [55–165]	ND	ND
N	5632[ICEA *ncr19*/*E*::mTn]^G^23 + pO/T-CDS5	PG2^P^ + pO/T	170 [103–237]	ND	ND
					
Complementation of *cds22* knockout ICEAs					
O	5632[ICEA *cds22*::mTn]^G^50 + pO/T-CDS22	PG2^P^ + pO/T	4 [1–7]	0	5
P	5632[ICEA *cds22*::mTn]^G^50 + pO/T	PG2^P^ + pO/T	2 [1–3]	0	5
Q	5632[ICEA *ncr19*/*E*::mTn]^G^23 + pO/T-CDS22	PG2^P^ + pO/T	40 [35–45]	1	1

a Mating experiments were performed with single clones grown and coincubated in SP4 medium containing tetracycline (2 µg/ml).

b PG2 ICE donors were generated upon mating with individual 5632 mutants and a PG2 clone carrying an enrofloxacin resistance tag (E); the mutant number refers to mutant ICEAs (gentamicin tagged; G) generated in 5632 by mTn mutagenesis or designates mutant ICEAs selected in PG2 upon mating with 5632 (Fig. 2; see also Table S2); plasmid constructions used for the complementation are indicated; the ICE donor in mating A to D differs from the study reported by Dordet-Frisoni et al. (7) by the site of ICEA integration in the chromosome of PG2 (chromosomal positions 135303 and 337636, respectively).

c The PG2 recipient cells were labeled with an mTn encoding resistance to puromycin (P) and transformed with the empty vector (pO/T) or the vector expressing CDS14 (pO/T-CDS14).

d The values shown were expressed as means ± standard deviations when the number of independent assays was ≥3 or as the average of results from two independent assays with each individual value in brackets; dually resistant colonies were selected by using a combination of gentamicin and puromycin; mating frequencies per singly resistant CFU are provided in Table S3.

e Doubly resistant colonies were genetically characterized to differentiate PG2 transconjugants from 5632 transconjugants that have acquired PG2 genomic materials by CTs (see Materials and Methods); the number of clones with a PG2 or 5632 genomic profile is indicated; NA, not applicable; ND, not determined.

f Selection of false-positive transconjugants (lacking one or the other resistance marker) with a frequency of <10^−9^ (detection limit, 1 × 10^−10^).

g PG2^E^[ICEA *cds14*::mTn]^G^28 was transformed with plasmid pO/T-CDS14bov expressing the M. bovis PG45 homologue of CDS14 (MBOVPG45_0187).

Interestingly, global alignment of CDS14 lipoprotein with its homologue in ICEB, the conjugative element occurring in the closely related M. bovis species, revealed 86.9% sequence similarity (Fig. S2). To test whether these differences might influence the conjugative transfer of ICEA in M. agalactiae, PG2^E^[ICEA *cds14*::mTn]^G^28 was transformed with plasmid pO/T-CDS14bov expressing the ICEB CDS14 lipoprotein. This plasmid was able to restore the conjugative properties of *cds14* knockout ICEA with a 2-fold reduction in mating frequency (Table 1, mating D). This suggests that one of the CDS14 functions is conserved between ICEA and ICEB.

Taken together, these results unveiled the critical role played by ICE-encoded surface lipoproteins in the exchange of genetic information within mycoplasma species and most likely across species.

### CDS5 expression from coresident ICEAs is a key factor for *cds5* knockout ICEA complementation.

In contrast to the *cds14* mutants that can be complemented by coresident ICEAs, or even by neighboring cells expressing CDS14, a large number of mutant ICEAs were unable to disseminate from 5632 to PG2 (Fig. 2; see also Table S2). Several of these mutant ICEAs were knocked out in genes whose products were not detected by proteomics (see below), raising the issue of whether the level of gene expression in coresident ICEAs can influence their cooperative behavior. To address this issue, complementation studies were performed using plasmid DNA constructs expressing CDS5.

While *cds5* knockout ICEAs have been shown to lose their capacity to disseminate from 5632 to PG2 (Fig. 2D, mutants 11 and 12), mating of 5632 complemented with *cds5* (plasmid pO/T-CDS5) with ICEA-negative PG2 recipient cells resulted in a ca. 10-fold increase in mating frequency (Table 1, matings E and G). Analysis of the mating progeny revealed that 64% to 78% of these transconjugants displayed a PG2 genomic profile, indicating conjugative transfer of the *cds5* knockout ICEA from 5632 to PG2. Transformation with the empty vector (plasmid pO/T) as a negative control resulted in no detectable event of transfer (Table 1, matings F and H). These data showed that *cds5* knockout ICEAs can be complemented in *trans*, at least partially, when *cds5* is expressed from the expression vector, while, paradoxically, coresident ICEAs were unable to restore the conjugative properties of *cds5* knockout ICEAs. Finally, these complementation studies allowed us to rule out any lethal effect resulting from integration of *cds5* knockout ICEAs in the PG2 chromosome.

The CDS5 is a putative membrane-bound hexamer with ATPase activity displaying some similarity to the TraG/VirD4 conjugative channel component found in the more classical bacteria (3). The formation of a hexameric structure by *cds5* products remains to be confirmed, but this multimeric organization may provide an alternative scenario for the inactive *cds5* knockout ICEAs in 5632. Indeed, mTn insertion in *cds5* could lead to the expression of truncated products interfering with the hexamer complex formation and thus inducing a dominant-negative effect on coresident ICEAs. To address this issue, truncated versions of *cds5* were cloned into the pO/T expression vector, leading to plasmids pO/T-CDS5N1, pO/T-CDS5N2, pO/T-CDS5C1, and pO/T-CDS5C2, expressing, respectively, CDS5 N- and C-terminal regions resulting from mTn insertion in *cds5* mutants 11 and 12 (Fig. S3). Transformation of 5632[ICEA *ncr19*/*E*::mTn]^G^23 (mTn inserted in *ncr19*/*E* with no influence on conjugation) with constructions carrying truncated forms of *cds5*, the full-length *cds5*, or the empty vector had no influence on mating efficacy (Table 1, matings I to N), indicating that the expression of CDS5 truncated products did not inhibit ICEA transfer and that the conjugation-deficient phenotype of *cds5* knockout mutants was not the result of a dominant-negative effect.

10.1128/mBio.00873-18.3 FIG S3  Plasmid constructions carrying *cds5* or truncated versions of *cds5*. Schematics illustrate plasmid constructions carrying full-length *cds5* (pO/T-CDS5) or truncated versions of *cds5* (pO/T-CDS5 N1, C1, N2, and C2). The truncated sequences are CDS5 N- and C-terminal regions resulting from mTn insertion in 5632[ICEA *cds5*::mTn]^G^11 and 5632[ICEA *cds5*::mTn]^G^12 (Fig. 2B; see also Table S2). Coding sequences were cloned downstream of the M. agalactiae lipoprotein P40 gene (MAG2410) promoter region (arrow). Download FIG S3, TIF file, 1.2 MB. Copyright © 2018 Baranowski et al. 2018 Baranowski et al. This content is distributed under the terms of the Creative Commons Attribution 4.0 International license.

### CDS22 expression is unable to *trans*-complement *cds22* knockout ICEAs.

A second series of complementation studies were performed with the *cds22* knockout ICEA mutant 5632[ICEA *cds22*::mTn]^G^50. That gene encodes a DDE recombinase that was previously shown to mediate ICEA excision and circularization (7). Transformation with pO/T-CDS22 did not increase mating frequency compared to the level seen with empty vector, and no PG2 transconjugants were identified upon analysis of the mating progeny (Table 1, matings O and P). Transformation of 5632[ICEA *ncr19*/*E*::mTn]^G^23 (mTn inserted in *ncr19*/*E* with no influence on conjugation) with pO/T-CDS22 or the empty vector had no or minimal influence on the mating frequencies (Table 1, matings Q and M). These results suggest that the *cds22* knockout ICEA cannot be complemented in *trans* either by coresident ICEAs or by a CDS22-expressing plasmid. This result is consistent with the longstanding observation that DDE transposases show a *cis* preference for their activities (20–22).

Taken together, these results illustrate the complex interactions taking place among coresident ICEAs in 5632 and elucidate some of the mechanisms underlying their noncooperative behavior.

### Protein expression profiles of PG2-ICEA mutants.

A previous study showed that three ICEA products in 5632, namely, CDS14 and, to a lesser extent, CDS17 and CDS30, are detectable by proteomic analysis under laboratory conditions (16). To further characterize ICEA expression in different genomic contexts, a proteomic analysis was conducted using a set of PG2 transconjugants that had acquired a mutated ICEA copy from 5632 (Fig. 3C; see also Table S4). Data revealed that up to 9 ICEA products, namely, CDSC, CDSD, CDS7, CDS15, CDS19, CDSE, CDS14, CDSF, and CDS30, were detected in PG2^T^[ICEA *ncr19*/*E*::mTn]^G^23 (mTn inserted in *ncr19*/*E* with no influence on conjugation). CDS17 was also detected in PG2^T^[ICEA *ncr19*/*E*::mTn]^G^23 but at levels below the cutoff value. CDSE, CDS14, and CDSF were not detected in PG2^T^[ICEA *cdsE*::mTn]^G^25, PG2^T^[ICEA *cds14*::mTn]^G^28, or PG2^T^[ICEA *cdsF*::mTn]^G^35, in all of which the corresponding genes are disrupted. Besides confirming the disruption of these genes, these data also indicate that mTn insertion in *cdsE* has no polar effect on the expression of the downstream *cds14* gene.

Interestingly, the data suggested that some ICEA loci might be downregulated to undetectable levels in the conjugation-deficient PG2^T^[ICEA *cds14*::mTn]^G^28 mutant. These corresponded to CDSC, CDSD, CDS7, and CDS19 detected in other mutants, whose genes are located upstream of *cds14*. Overall, the PG2 ICEA mutants that were tested here and disrupted in identified coding genes, versus *ncr19*/*E*, had a simplified ICEA protein expression profile.

## DISCUSSION

Since their discovery in mycoplasma species of the hominis phylogenetic group, MICEs have been found broadly distributed across the members of *Mollicutes* (2–6, 8) and their pivotal role in HGTs is emerging (7, 12, 13). Taking advantage of the M. agalactiae ICE prototype, this report provides the first functional analysis of MICE factors involved in conjugative transfer. Because MICEs, such as ICEA, are often found in multiple copies, this report points toward their complex interplay in the mycoplasma host environment.

### The functional ICEA backbone.

The minimal ICEA chassis conferring conjugative properties to M. agalactiae was identified by random transposon mutagenesis. Of the 23 genes reported in ICEA, 17 were found to be disrupted by the insertion of an mTn and 13 were found essential for self-dissemination, since a single mTn insertion in any of these regions abrogated the conjugative properties of M. agalactiae. These data point toward the minimal ICEA machinery being composed of (i) a cluster of 7 proteins with predicted transmembrane domains that most likely represents a module associated with the conjugative channel (CDS5 to CDS19), (ii) a surface-exposed lipoprotein (CDS14), (iii) a putative partitioning protein (CDSG), (iv) a DDE transposase (CDS22), and (v) several other proteins with no predicted function (CDS1, CDSA, CDSC, and CDS30). The conjugation-deficient phenotype of the ICEA mutants is unlikely to be the result of a polar effect since (i) mTn insertions were identified in close proximity to essential ICEA regions with no influence on conjugation (*cds1*, *cds14*, *cds30*, and, to a lesser extent, *cdsA* and *cdsG*); (ii) *cds14* knockout ICEAs can be complemented in *trans*; (iii) ICEA mutants that have the putative channel module disrupted by an mTn inserted in *cds5* can be plasmid complemented; and (iv) mTn insertions in the *cdsE*-*cdsH* region have no influence on protein expression from surrounding genes. Whether additional essential ICEA functions may be encoded by several of the 6 short genes (0.20 to 0.65 kb) with no mTn insertion remains to be further investigated.

The minimal ICEA chassis was consistent with the conservation of *cds5*, *cds17*, *cds19*, and *cds22* across documented ICEs of ruminant mycoplasma species (8) and with the occurrence of *cds1*, *cds14*, and *cds16* at very similar locations in a majority of MICEs (2, 4–6, 23). Interestingly, the conjugative properties of M. agalactiae were also abrogated by mTn insertion in NCRs, raising questions regarding the presence of regulatory elements and/or key motifs, such as an *oriT*, in these regions. The identification of the occurrence of such sequences in the NCR1/A (1,238 nucleotides) is supported by the identification of a hairpin motif (TGGCTCAT-N_5_-ATGAGCCA) at positions 2046 to 2066 (S. Torres-Puig, personal communication). Whether mTn insertion in NCRs may influence the expression surrounding ICEA regions is unknown.

Accessory ICEA functions were found associated with only 4 genes, namely, *cds11*, *cdsE*, *cdsF*, and *cdsH*. These accessory functions are all encoded by genes within a 6.4-kb ICEA region spanning *cdsE* to *cdsH*, with the exception of *cds11*, which belongs to a cluster of 6 genes (*cdsA* to *cdsD*) located upstream from the putative channel module (Fig. 1). Although dispensable, an important reduction of the mating frequency was observed for several mutants with an mTn inserted in these ICEA regions (Fig. 2C). For PG2^T^[ICEA *cds11*::mTn]^G^7 (Fig. 2A, mutant 7), this reduction was not influenced by the position of the mutant ICEA in the PG2 chromosome. Finally, BLASTP analyses with CDSE revealed significant (≥90%) similarity to a putative prophage gene product found in the chromosomes of PG2 (MAG6440) and 5632 (MAGa7400). The presence of this chromosomal *cdsE* homologue is puzzling, and its role in ICEA transfer remains to be confirmed.

### A minimal genome able to cope with multiple ICEA copies.

Unlike classical ICEs, ICEA has no preferential insertion specificity and multiple copies can be found at different loci of the host chromosome. This raised questions regarding their maintenance in the small mycoplasma genomes and the deleterious effect that can be associated with their random insertion, in particular, because they were found within coding sequences (7, 16). Whether ICEA may confer any advantage *in vivo* is unknown, but PG2 ICEA transconjugants displayed reduced fitness under laboratory conditions (unpublished data). Many bacterial ICEs and some prokaryotic transposable elements carry cargo genes implicated in accessory functions, such as antibiotic resistance, which confer a selective advantage to their host (1). Such cargo genes have never been reported in MICEs, but ICE-mediated CTs likely contribute to the acquisition of new phenotypic traits upon chromosomal exchanges.

### Backup functions associated with coresident ICEAs.

Our data suggest that coresident ICEAs are able to cooperate by complementing essential functions in mutant ICEAs. This was shown by using *cds14* knockout ICEAs, which can self-disseminate in the context of strain 5632 but not in the context of PG2, which contains no additional ICEA copy. The complementation of *cds14* by coresident ICEAs was further confirmed by the constitutive expression of the CDS14 lipoprotein in 5632 mutants with 1 of the 3 ICEA copies knocked out in *cds14* but not in PG2 cells that had acquired a *cds14* knockout ICEA. Remarkably, this cooperative behavior was found to extend to neighboring cells, since transformants of ICEA-negative cells containing a plasmid vector expressing CDS14 were able to complement *cds14* knockout ICEAs in neighboring cells. Besides providing the first example of ICE complementation by neighboring cells, this result has deep implications for the dissemination of MICEs within and across mycoplasma species. This idea was further supported by complementation studies showing that the CDS14 lipoprotein in M. agalactiae can be substituted by its homologue in M. bovis ICEB and by the results of our previous study showing ICEA-mediated CTs between M. agalactiae and M. bovis (12).

Interestingly, the cooperative behavior documented with *cds14* knockout ICEAs did not extend to other critical ICEA regions. Complementation studies with *cds22* knockout ICEAs confirmed that several critical ICEA functions can be associated with *cis*-acting elements that cannot be complemented by coresident ICEAs. However, studies with *cds5* mutant ICEAs suggested that interactions among coresident ICEAs can be more complex. Indeed, *cds5* knockout ICEAs can be *trans*-complemented upon transformation with a CDS5-expressing plasmid but not by coresident ICEAs. Since ICEA transfer from 5632 to PG2 occurs at only a low frequency, ICEA activation is expected to be a rare event. It is thus reasonable to speculate that only one of the three chromosomal ICEA copies in 5632 can be stochastically activated. This hypothesis provides a simple scenario to explain the lack of complementation of *cds5* mutants by coresident ICEAs, since this component of the mating channel is expected to be expressed only upon ICEA activation. It is further supported by proteomic analysis showing a simplified ICEA protein expression profile that contrasted with the constitutive expression of the CDS14 surface lipoprotein.

### Conclusions.

**FIG 4:**
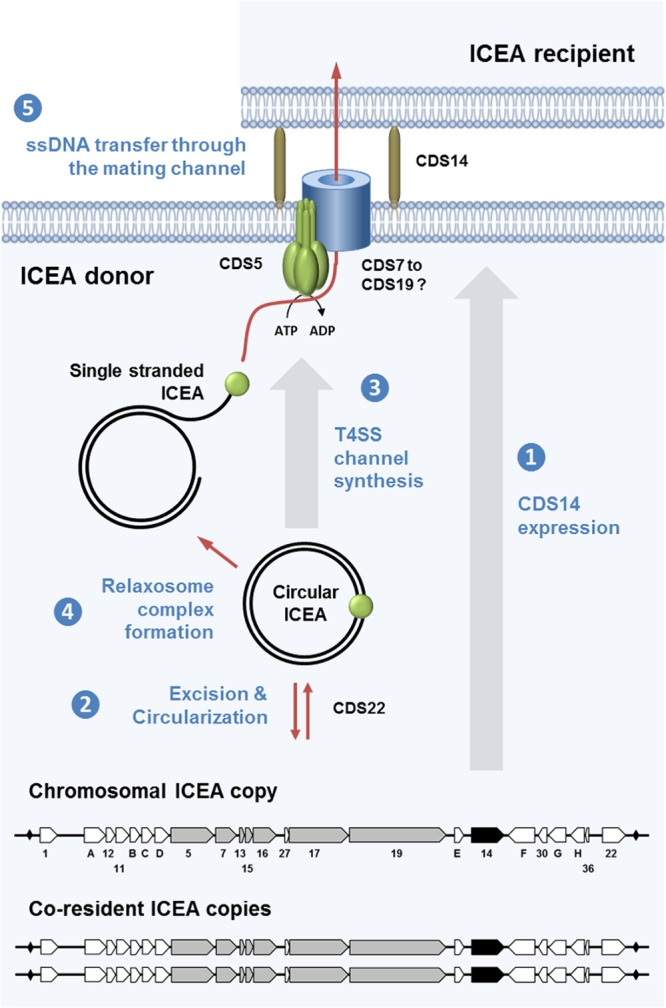
Overview of conjugative ICE transfer in M. agalactiae. This schematic illustrates the 5 key steps in ICEA transfer based on current knowledge in other bacteria (1, 18). Under normal conditions, ICEA copies are found integrated into the host chromosome and most ICEA genes are not expressed. Among the few proteins expressed by chromosomal ICEAs is the CDS14 lipoprotein, which is surface exposed and plays a critical role in initiating the conjugative process (step 1). When ICEA gene expression is induced, under specific cellular conditions or stochastically, the *cis*-acting DDE transposase is produced and one of the three ICEA copies excises from the chromosome and forms a circular double-stranded DNA (dsDNA) molecule (step 2). ICEA circularization induces the expression of the conjugative module, whose products assemble into the mating pore, a simplified form of type IV secretion system (T4SS) found in more-complex bacteria (step 3). A protein complex known as a relaxosome recognizes the origin of transfer (*oriT*) on the circular ICEA, and a relaxase generates a linear single-stranded DNA (ssDNA) by nicking the ICEA DNA (step 4). Finally, the relaxosome complex interacts with the TraG-like (VirD4 homologue) energetic component found at the inner side of the membrane that facilitates the transfer of the ssDNA bound to the relaxase through the mating channel (step 5). Once in the recipient strain, the ICEA recircularizes, becomes doubly stranded, and integrates randomly into the host chromosome. The minimal functional ICEA encompasses 80% of the coding sequence and includes a gene cluster (*cds5* to *cds19*, encoding proteins with transmembrane domains) that most likely represents a module associated with the conjugative channel. Additional essential ICEA determinants included the CDS14 surface lipoprotein, the CDSG putative partitioning protein, and the DDE transposase (CDS22), together with several proteins of unknown function (CDS1, CDSA, CDSC, and CDS30).

The results generated in the present study were combined with current knowledge to propose the first working model of horizontal ICE dissemination in mycoplasmas, including cooperation among coresident ICEs (Fig. 4). These data, together with the large collection of ICEA mutants generated in this study, pave the way for future studies aiming at deciphering ICE-mediated CTs within and among mycoplasma species. These simple organisms also provide a valuable experimental frame to decipher the mechanisms of DNA exchange in more-complex bacteria in association with this new category of mobile elements.

## MATERIALS AND METHODS

### Mycoplasmas and culture conditions.

M. agalactiae strains PG2 and 5632 have been previously described (14, 16), and the sequence of each genome is available in databases (GenBank accession numbers CU179680.1 and FP671138.1, respectively). These two strains differ in their ICE content, with strain 5632 having three almost identical chromosomal copies of ICEA (ICEA-I, ICEA-II, and ICEA-III), while strain PG2 contains only a severely degenerated, vestigial ICE (14, 16). M. agalactiae was grown at 37°C in SP4 medium supplemented with 500 µg/ml cephalexin (Virbac). When needed, gentamicin (50 µg/ml), tetracycline (2 µg/ml), and puromycin (10 µg/ml), alone or in combination, were added to the medium. Due to their small cell size and colony size, growth of mycoplasmas cannot be monitored by optical density. Mycoplasma titers were thus determined based on colony counts on solid media after 4 to 7 days of incubation at 37°C performed by the use of a binocular stereoscopic microscope (19).

### Transposon mutagenesis and genetic tagging of mycoplasmas with antibiotic markers.

A similar approach was used for transposon mutagenesis and genetic tagging of M. agalactiae. Selective antibiotic markers were introduced randomly in the mycoplasma genome as previously described by transforming mycoplasma cultures with plasmid pMT85 or its derivatives (7, 19, 24). The pMT85 carries a minitransposon (mTn) derived from the Tn*4001* gentamicin resistance transposon. The transposase gene (*tnpA*) is located outside the mTn to prevent re-excision events once it is inserted in the host chromosome (19). Two derivatives, pMT85-Tet and pMT85-Pur, were constructed as previously described by replacing the gentamicin resistance gene with a tetracycline resistance marker and a puromycin resistance marker, respectively (7).

### PCR-based screening of mycoplasma mutant library.

A set of 19 oligonucleotides spanning the whole ICEA region (see Table S5 in the supplemental material) was used to develop a PCR-based screening of the mutant library and to identify 5632 mutants with an mTn inserted within ICEA regions. Each ICEA-specific primer was used in combination with the SG5 transposon-specific oligonucleotide priming at both inverted repeats (IRs) that define the extremities of the integrated transposon (Table S5). PCR ampliﬁcations were performed according to the recommendations of the *Taq* DNA polymerase supplier (New England Biolabs). For each mutant, the position of the mTn insertion in the M. agalactiae chromosome was determined by sequencing the junction between M. agalactiae genomic DNA and the 5′ end or 3′ end of the transposon using oligonucleotides SG6_3pMT85E (speciﬁc to the 5′ end of the gentamicin-tagged version of the mTn), SG9pMM21-7mod (speciﬁc to the 5′ end of the tetracycline-tagged version of the mTn), and EB8 (speciﬁc to the 3′ end of all mTn constructions) as primers (Table S5). Genomic DNA sequencing was performed at the GeT-Purpan genomic platform (Toulouse, France). The distribution of mTn insertions among the three ICEA copies was determined by long-range PCR amplifications (Expand long-template PCR system; Roche Life Sciences) using mTn-specific primers and a panel of oligonucleotides corresponding to genomic DNA regions surrounding each ICEA locus (Table S5).

10.1128/mBio.00873-18.8 TABLE S5  Oligonucleotides used in the present study. Download TABLE S5, XLSX file, 0.02 MB. Copyright © 2018 Baranowski et al. 2018 Baranowski et al. This content is distributed under the terms of the Creative Commons Attribution 4.0 International license.

### Mycoplasma mating experiments and genetic characterization of transconjugant progenies.

Mating experiments were conducted as described previously by coincubation of ICE-positive and ICE-negative cells (7). Mycoplasma growth may considerably vary from batch to batch using the rich SP4 medium, which contains serum and yeast extract. To reduce potential bias in the comparisons of the mating frequencies observed between experiments, a single batch of medium was used in this study. Cultures of donor and recipient mycoplasmas (10^9^ CFU) were mixed in a 1:1 ratio (matings PG2 ICEA × PG2) or a 1:10 ratio (matings 5632 × PG2) to increase the chances of recovering PG2 ICEA transconjugants. The mating frequency was calculated by dividing the number of doubly resistant colonies obtained on selective solid media by the number of mycoplasma colonies obtained on nonselective media. M. agalactiae transconjugants were characterized by PCR amplification using genomic DNA prepared from individual colonies (7). The presence of antibiotic resistance genes and ICEA in transconjugants was confirmed by using specific oligonucleotides (Table S5). The nature of the genetic backbone was addressed by using a set of primer pairs that covers the M. agalactiae genome and produces PCR fragments specific to 5632 or PG2 (Table S5), as previously described (7, 12).

### DNA constructs for protein expression in mycoplasmas.

Protein expression in M. agalactiae was performed as previously described by using plasmid p20-1miniO/T (designated "pO/T" in the present study) (19, 25). Briefly, mycoplasma coding sequences were cloned downstream of the lipoprotein P40 gene (MAG2410) promoter region. These two regions were assembled by PCR ampliﬁcation using overlapping primers (Table S5). The resulting PCR product was cloned into pGEM-T Easy (Promega) before subcloning at the NotI site of the pO/T was performed. PCRs were performed using Phusion high-fidelity DNA polymerase (New England Biolabs). DNA constructions were verified by DNA sequencing and introduced in M. agalactiae by transformation, as previously described (19).

### Proteomic analyses and immunodetection of ICEA products.

M. agalactiae grown under normal and mating growth conditions was subjected to proteomic analyses. Cells were collected by centrifugation of mycoplasma cultures (8,000 × *g*), washed, and resuspended in Dulbecco’s phosphate-buffered saline (DPBS). Proteins were separated by one-dimensional (1D) SDS-PAGE, and gel sections were subjected to trypsin digestion. Peptides were further analyzed by nano liquid chromatography coupled to a nanospray Q-Exactive hybrid quadruplole-Orbitrap mass spectrometer (Thermo Scientific). Peptides were identified as previously described by using a database consisting of M. agalactiae strain 5632 entries (26). ICEA products were detected by specific antiserums on Western and colony blots (25, 27). Triton-X114-soluble proteins were extracted from M. agalactiae as previously described (28). The anti-CDS14 lipoprotein rabbit serum was produced by animal immunization with a recombinant CDS14 protein (pMAL protein fusion and purification system; New England Biolabs). A sheep serum raised against the M. agalactiae P80 surface antigen was used as a control (25). Western and colony blotting was developed by using swine anti-rabbit or rabbit anti-sheep immunoglobulin G conjugated to horseradish peroxidase (DAKO) and 4-chloro-naphthol substrate or SuperSignal West Dura extended-duration substrate (Thermo Scientific).
