# Supplementary material for: The Integrative Conjugative Element (ICE) of Mycoplasma agalactiae: Key Elements Involved in Horizontal Dissemination and Influence of Coresident ICEs
Source: mBio. 2018 Jul 3;9(4):e00873-18. doi: 10.1128/mBio.00873-18 (PMC6030558; doi:10.1128/mBio.00873-18)
Supplement: TABLE S1 [file mbo004183966st1.docx]

**Table S1. Relevant features of ICEA products.**

| CDS | UniProt ^a^ | Putative conserved domains ^b^ | InterPro ^c^ |
| --- | --- | --- | --- |
| 1 | D3VQB9 | - | Winged helix-like DNA-binding domain superfamily (IPR036388) |
| A | D3VQC0 | - | - |
| 12 | D3VQV1 | - | Nucleic acid-binding, OB-fold (IPR012340)  Primosome PriB/single-strand DNA-binding (IPR000424) |
| 11 | D3VQC2 | - | - |
| B | D3VQC3 | - | - |
| C | D3VQC4 | - | - |
| D | D3VQC5 | - | - |
| 5 | D3VQC6 | TrwB_AAD_bind Superfamily, type IV secretion-system coupling protein DNA-binding domain (cl26533)  TraG_VirD4 domain family, TraG/TraD/VirD4 family are bacterial conjugation proteins involved in type IV secretion (cd01126)  P-loop_NTPase Superfamily, P-loop containing nucleoside triphosphate hydrolases (cl21455) | P-loop containing nucleoside triphosphate hydrolase (IPR027417)  T4SS protein TraG/VirD4 (IPR003688) |
| 7 | D3VQC7 | - | - |
| 13 | D3VQC8 | - | - |
| 15 | D3VQC9 | - | - |
| 16 | D3VQW0 | - | - |
| 27 | D3VQD2 | - | - |
| 17 | D3VQD3 | VirB4 Superfamily, type IV secretory pathway, VirB4 component (cl26286)  AAA Superfamily, ATPases associated with a variety of cellular activities (cl28181) | P-loop containing nucleoside triphosphate hydrolase (IPR027417) |
| 19 | D3VQD4 | - | - |
| E | D3VQD5 | DUF4065 Superfamily, protein of unknown function (cl01445) | - |
| 14 | D3VQD6 | - | - |
| F | D3VQW5 | DUF2130 Superfamily, uncharacterized protein conserved in bacteria (cl26585) | - |
| 30 | D3VQD8 | - | - |
| G | D3VQW7 | P-loop_NTPase Superfamily, P-loop containing Nucleoside Triphosphate Hydrolases (cl21455)  CbiA Superfamily, CobQ/CobB/MinD/ParA nucleotide binding domain (cl27521)  ParA, conserved family of bacterial proteins implicated in chromosome segregation (cd02042)  PRK13869 Superfamily, plasmid-partitioning protein RepA; provisional (cl27523) | P-loop containing nucleoside triphosphate hydrolase (IPR027417) |
| H | D3VRE6 | MethyltransfD12 Superfamily, D12 class N6 adenine-specific DNA methyltransferase (cl23779) | SAM-dependent methyltransferase (IPR029063)  D12 class N6 adenine-specific DNA methyltransferase (IPR012327) |
| 36 | - | - | - |
| 22 | D3VQW9 | - | - |

^a^ UniProtKB/TrEMBL entry; ^b^ Putative conserved domains were identified by blastp (E value < e10-4); homology searches were performed against the NCBI Protein Reference Sequence database (Refseq_protein); ^c^ InterPro classification of ICEA protein sequences into families.
