# Supplementary material for: The Integrative Conjugative Element (ICE) of Mycoplasma agalactiae: Key Elements Involved in Horizontal Dissemination and Influence of Coresident ICEs
Source: mBio. 2018 Jul 3;9(4):e00873-18. doi: 10.1128/mBio.00873-18 (PMC6030558; doi:10.1128/mBio.00873-18)
Supplement: TABLE S2 [file mbo004183966st2.docx]

**Table S2.** Mutant ICEAs generated in *M. agalactiae* strain 5632 ^a^.

| **ICEA mutant**  **number** | **ICEA knock-out region** | **MTn Insertion site** ^b^ | **MTn orientation (genome/CDS)** | **Relative CDS position** | **Mutant-ICEA copy number** ^c^ | **Screening strategy** | |
| --- | --- | --- | --- | --- | --- | --- | --- |
|  |  |  |  |  |  | **Mating** ^d^  **(clones)** | **PCR** |
| 1 | *cds1* | 00464 | - / - | 0.43 | III | - | + |
| 2 | *ncr1/A* | 01243 | + / n.a. | n.a. | II | - | + |
| 3 | *ncr1/A* | 01342 | - / n.a. | n.a. | n.d. | + (2) | n.d. |
| 4 | *ncr1/A* | 01349 | + / n.a. | n.a. | III | - | + |
| 5 | *ncr1/A* | 01734 | - / n.a. | n.a. | n.d. | + (1) | n.d. |
| 6 | *cdsA* | 02653 | - / - | 0.49 | I | - | + |
| 7 | *cds11* | 03816 | - / - | 0.33 | I | + (1) | + |
| 8 | *ncrB/C* | 04835 | + / n.a. | n.a. | I | - | + |
| 9 | *cdsC* | 04853 | - / - | 0.02 | II | - | + |
| 10 | *ncrD/5* | 06119 | - / n.a. | n.a. | III | - | + |
| 11 | *cds5* | 06356 | - / - | 0.08 | I | - | + |
| 12 | *cds5* | 06953 | - / - | 0.37 | II | - | + |
| 13 | *cds7* | 08861 | - / - | 0.61 | II | - | + |
| 14 | *cds15* | 09667 | + / + | 0.05 | II | - | + |
| 15 | *cds15* | 09928 | + / + | 0.77 | II | - | + |
| 16 | *cds16* | 11000 | + / + | 0.91 | II | - | + |
| 17 | *cds16* | 11018 | - / - | 0.93 | III | - | + |
| 18 | *ncr16/27* | 11183 | - / n.a. | n.a. | III | - | + |
| 19 | *cds17* | 13303 | - / - | 0.59 | III | - | + |
| 20 | *cds19* | 16500 | - / - | 0.45 | n.d. | - | + |
| 21* | *ncr19/E* | 19130 | + / n.a. | n.a. | n.d. | + (1) | n.d. |
| 22 | *ncr19/E* | 19171 | - / n.a. | n.a. | n.d. | + (1) | n.d. |
| 23 | *ncr19/E* | 19198 | + / n.a. | n.a. | I | + (6) | + |
| 24* | *cdsE* | 19503 | + / + | 0.25 | n.d. | + (2) | n.d. |
| 25 | *cdsE* | 19649 | - / - | 0.58 | II | + (1) | + |
| 26 | *cdsE* | 19690 | - / - | 0.67 | n.d. | + (5) | n.d. |
| 27 | *ncrE/14* | 19856 | + / n.a. | n.a. | I | + (4) | n.d. |
| 28 | *cds14* | 20179 | - / - | 0.01 | I | + (6) | + |
| 29 | *cds14* | 20773 | - / - | 0.39 | n.d. | + (1) | n.d. |
| 30 | *cds14* | 21225 | + / + | 0.68 | n.d. | + (1) | n.d. |
| 31 | *cds14* | 21237 | + / + | 0.69 | n.d. | + (2) | n.d. |
| 32* | *cds14* | 21566 | - / - | 0.89 | n.d. | + (1) | n.d. |
| 33 | *cds14* | 21662 | + / + | 0.95 | n.d. | + (1) | n.d. |
| 34 | *cdsF* | 22176 | - / + | 0.75 | n.d. | + (2) | n.d. |
| 35 | *cdsF* | 22192 | - / + | 0.74 | II | + (2) | + |
| 36 | *cdsF* | 22442 | - / + | 0.54 | n.d. | + (2) | n.d. |
| 37 | *cdsF* | 22598 | - / + | 0.41 | I | + (1) | + |
| 38 | *cdsF* | 22838 | - / + | 0.22 | III | + (4) | + |
| 39 | *cds30* | 23493 | - / + | 0.43 | II | - | + |
| 40 | *cdsG* | 23769 | + / - | 0.96 | II | - | + |
| 41 | *cdsG* | 24417 | + / - | 0.19 | III | - | + |
| 42 | *cdsH* | 24773 | + / - | 0.99 | III | + (2) | + |
| 43 | *cdsH* | 24800 | + / - | 0.95 | III | + (1) | + |
| 44 | *cdsH* | 25221 | + / - | 0.32 | I | + (3) | + |
| 45 | *cdsH* | 25420 | - / + | 0.03 | n.d. | + (2) | n.d. |
| 46* | *cdsH* | 25433 | + / - | 0.01 | n.d. | + (2) | n.d. |
| 47 | *ncr36/22* | 25967 | - / n.a. | n.a. | I | + (1) | + |
| 48 | *cds22* | 26355 | +/+ | 0.12 | II | - | + |
| 49 | *cds22* | 26365 | +/+ | 0.13 | III | - | + |
| 50 | *cds22* | 26672 | +/+ | 0.40 | III | - | + |
| 51 | *cds22* | 27046 | +/+ | 0.73 | II | - | + |
|  |  |  |  | **Total** |  | **27** | **35** |

^a^ Transposon mutagenesis was performed by using a mTn encoding resistance to gentamicin or tetracycline (*); *cds*: coding sequence; *ncr*: non-coding region; n.a. : not applicable; n.d. : not determined. ^b^ The genomic positions all refer to ICEA copy number III (NCBI Reference Sequence NC_013948.1; genomic region 560518 to 588021). ^C^ Distribution of mTn insertions among the three ICEA copies of the 5632 mutants generated by transposon mutagenesis. ^d^ The number of PG2 transconjugants sharing a mTn inserted at the same chromosomal position is indicated in parenthesis.
