# Supplementary material for: The Integrative Conjugative Element (ICE) of Mycoplasma agalactiae: Key Elements Involved in Horizontal Dissemination and Influence of Coresident ICEs
Source: mBio. 2018 Jul 3;9(4):e00873-18. doi: 10.1128/mBio.00873-18 (PMC6030558; doi:10.1128/mBio.00873-18)
Supplement: TABLE S3 [file mbo004183966st3.docx]

**Table S3.1.** Mating frequencies per single-resistant CFUs in mating experiments involving PG2 ICEA and PG2 as partners

| **Mutant ICEA number ^a^** | **ICE Donor ^b^** | **ICE Recipient** | **Mating frequency (x 10^-8^) per ^c^** | |
| --- | --- | --- | --- | --- |
|  |  |  | **Gm-resistant colonies** | **Puro-resistant colonies** |
| 7a | PG2^T^[ICEA *cds11*::mTn]^G^7a | PG2^P^ | 36 ± 19 | 3 ± 1 |
| 7b | PG2^T^[ICEA *cds11*::mTn]^G^7b | PG2^P^ | 15 ± 7 | 1 ± 0.5 |
| 23 | PG2^T^[ICEA *ncr19/E*::mTn]^G^23 | PG2^P^ | 596 ± 149 | 127 ± 18 |

^a^ The mutant ICEA number refers to Figure 2C (mating PG2 ICEA x PG2); PG2 mutants 7a and 7b are sharing the same mutant ICEA integrated at different chromosomal sites (genomic positions 395291 and 433901 for mutants 7a and 7b, respectively). ^b^ Antibiotic-tags are indicated (T, tetracycline; G, gentamicin; P, puromycin). ^c^ The values shown are the means ± standard deviation of at least 3 independent assays; due to the occurrence of both ICE and chromosomal transfer during mating, Gm-resistant colonies include (i) cells having the parental PG2^T^[ICEA]^G^ genotype, (ii) PG2^P^-cells having received a Gm-labelled ICE, and (iii) PG2^T^[ICEA]^G^ cells having received chromosomal fragments from PG2^P^ that may or may not contain the puromycin marker. Similarly, Puro-resistant colonies include (i) the PG2^P^ parent cells, (ii) PG2^P^-cells having received a Gm-labelled ICE, and (iii) PG2^T^[ICEA]^G^ cells having received chromosomal fragments from PG2^P^ that contain the puromycin marker.

**Table S3.2.** Mating frequencies per single-resistant CFUs in mating experiments involving 5632 and PG2 as partners

| **Mutant ICEA number ^a^** | **ICE Donor ^b^** | **ICE Recipient** | **Mating frequency (x 10^-8^) per ^c^** | |
| --- | --- | --- | --- | --- |
|  |  |  | **Gm-resistant colonies** | **Tet-resistant colonies** |
| 11 | 5632[ICEA *cds5*::mTn]^G^11 | PG2^T^ | 22 [4-39] | 7 [1-12] |
| 12 | 5632[ICEA *cds5*::mTn]^G^12 | PG2^T^ | 12 [1-23] | 4 [0.4-8] |
| 23 | 5632[ICEA *ncr19/E*::mTn]^G^23 | PG2^T^ | 378 [230-526] | 107 [84-129] |

^a^ The mutant ICEA number refers to Figure 2D (mating 5632 x PG2). ^b^ Antibiotic-tags are indicated (G, gentamicin; T, tetracycline). ^c^ The values shown are the average of two independent assays with individual values in brackets; due to the occurrence of both ICE and chromosomal transfer during mating, the Gm-resistant colonies include not only (i) the parental 5632[ICEA]^G^ and (ii) the PG2^T^ cells having received a Gm-labelled ICE, but also (iii) the 5632[ICEA]^G^ cells having lost unlabeled ICE copies and (iv) the 5632[ICEA]^G^ cells having received chromosomal fragments from PG2^T^ that may or may not contain the tetracycline marker. Similarly, Tet-resistant colonies include (i) the PG2^T^ parent cells, (ii) PG2^T^-cells having received a Gm-labelled ICE (iii) PG2^T^-cells having received unlabeled ICE copies, and (iv) 5632[ICEA]^G^ cells having received chromosomal fragments from PG2^T^ that contain the tetracycline marker.

**Table S3.3.** Mating frequencies per single-resistant CFUs in mating experiments involving *cds14* and *cds5* knock-out ICEAs

| **Mating** ^a^ | **ICE Donor ^b^** | **ICE Recipient** | **Mating frequency (x 10^-8^) per ^c^** | | | |
| --- | --- | --- | --- | --- | --- | --- |
|  |  |  | **Gm-resistant colonies** | **Puro-resistant colonies** | |  |
| ***Complementation of cds14 knock-out ICEAs*** | |  |  |  |  |  |
| A | PG2^E^[ICEA *cds14*::mTn]^G^ + pO/T-CDS14 | PG2^P^ + pO/T | 302 ± 166 | 623 ± 251 | |  |
| B | PG2^E^[ICEA *cds14*::mTn]^G^ + pO/T | PG2^P^ + pO/T | n.a. | n.a. | |  |
| C | PG2^E^[ICEA *cds14*::mTn]^G^ + pO/T | PG2^P^ + pO/T-CDS14 | 17 ± 9 | 20 ± 13 | |  |
| D | PG2^E^[ICEA *cds14*::mTn]^G^ + pO/T-CDS14bov | PG2^P^ + pO/T | 221 [108-334] | 380 [177-583] | |  |
| ***Expression of CDS5 truncated products*** | |  |  |  |  |  |
| I | 5632[ICEA *ncr19/E*::mTn]^G^23 + pO/T-CDS5N1 | PG2^P^ + pO/T | 269 [147-390] | 271 [90-452] | |  |
| J | 5632[ICEA *ncr19/E*::mTn]^G^23 + pO/T-CDS5C1 | PG2^P^ + pO/T | 613 [171-1055] | 540 [457-623] | |  |
| K | 5632[ICEA *ncr19/E*::mTn]^G^23 + pO/T-CDS5N2 | PG2^P^ + pO/T | 306 [174-437] | 293 [150-436] | |  |
| L | 5632[ICEA *ncr19/E*::mTn]^G^23 + pO/T-CDS5C2 | PG2^P^ + pO/T | 384 [64-704] | 207 [184-230] | |  |
| M | 5632[ICEA *ncr19/E*::mTn]^G^23 + pO/T | PG2^P^ + pO/T | 196 [79-312] | 197 [196-197] | |  |
| N | 5632[ICEA *ncr19/E*::mTn]^G^23 + pO/T-CDS5 | PG2^P^ + pO/T | 540 [143-936] | 378 [221-535] | |  |

^a^ The letter refers to mating experiments A to Q (Table 1). ^b^ Antibiotic-tags are indicated (E, enrofloxacin; G, gentamicin; P, puromycin). ^c^ The values shown are the means ± standard deviation when the number of independent assays was ≥ 3, or the average of two independent assays with individual values in brackets; n.a.: not applicable (no transconjugant selected); as detailed in Tables S3.1 and S3.2, Gm-resistant and Puro-resistant colonies are complex populations resulting from ICE and chromosomal transfer during mating.
