# Supplementary figures and images for: The Integrative Conjugative Element (ICE) of Mycoplasma agalactiae: Key Elements Involved in Horizontal Dissemination and Influence of Coresident ICEs
Source: mBio. 2018 Jul 3;9(4):e00873-18. doi: 10.1128/mBio.00873-18 (PMC6030558; doi:10.1128/mBio.00873-18)

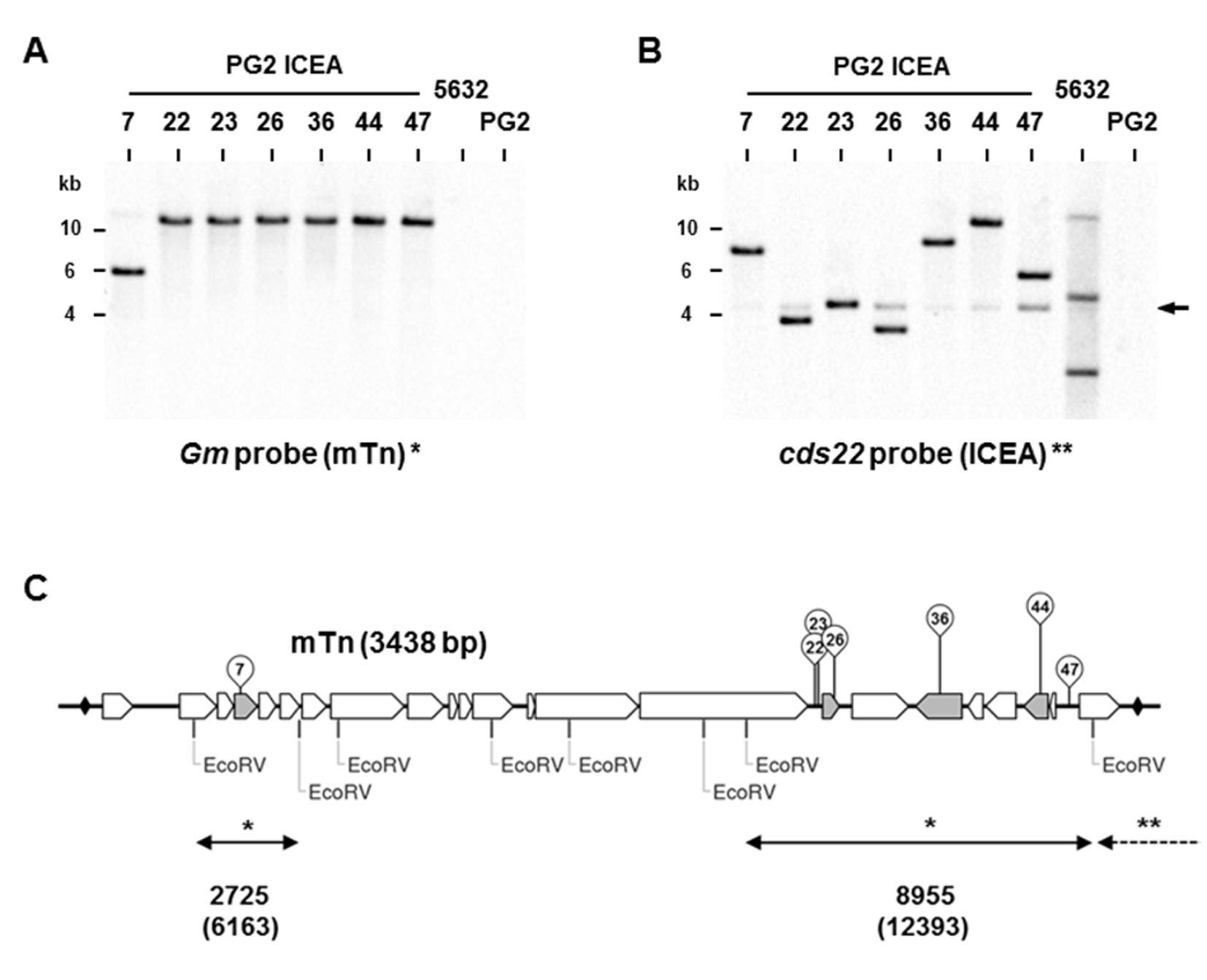

Supplement: FIG S1 [file mbo004183966sf1.tif]

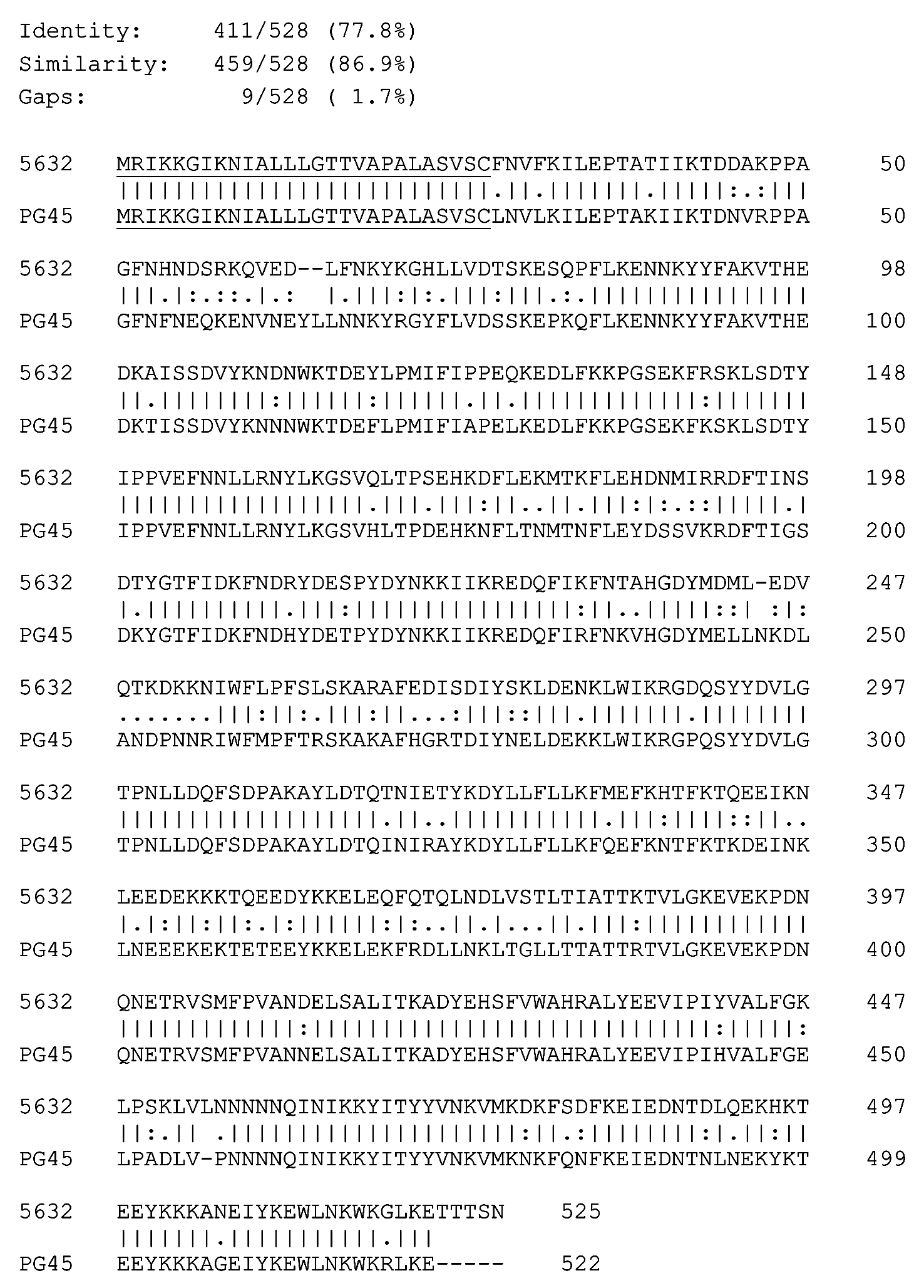

Supplement: FIG S2 [file mbo004183966sf2.tif]

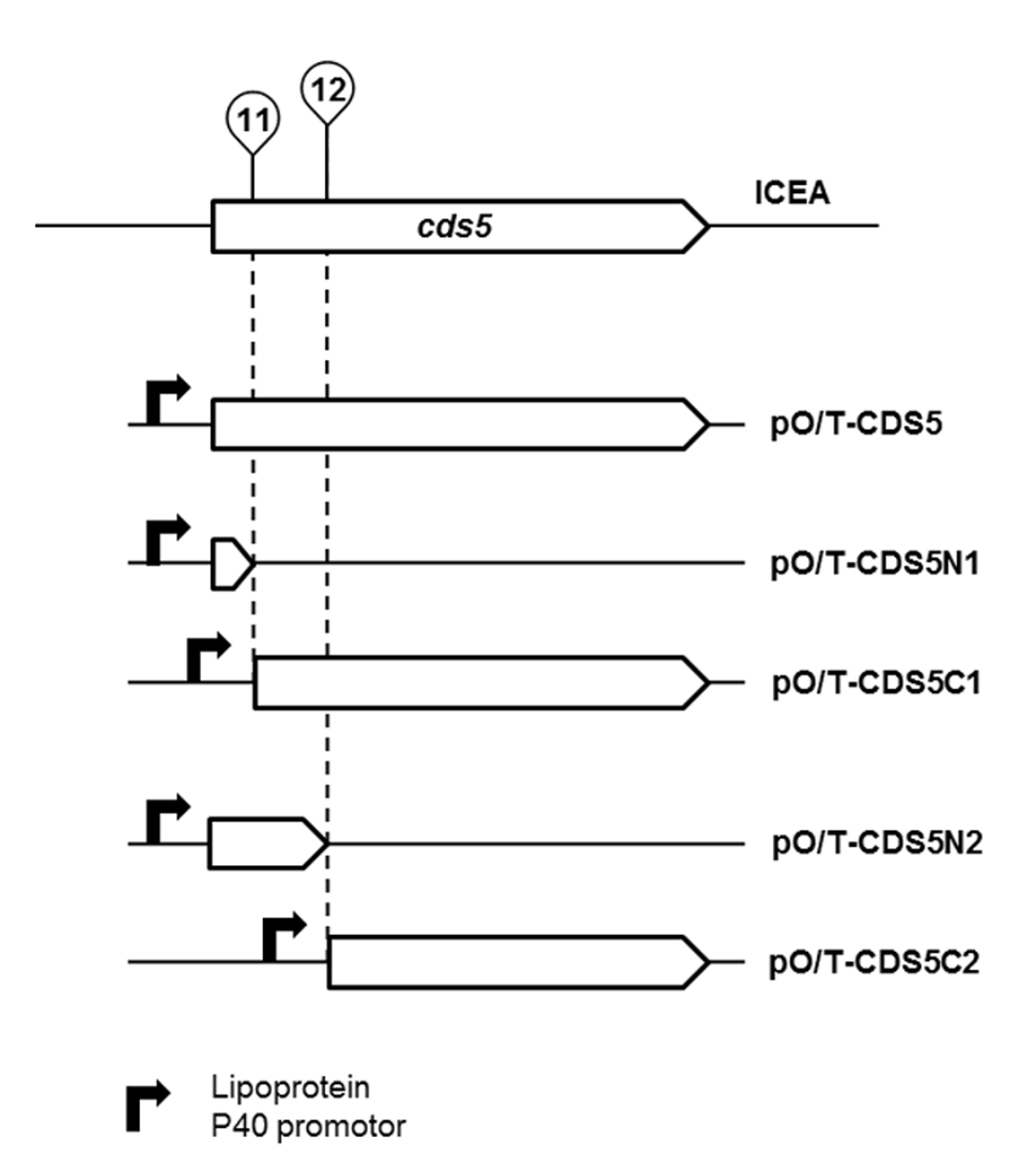

Supplement: FIG S3 [file mbo004183966sf3.tif]
