# Supplementary material for: The Integrative Conjugative Element (ICE) of Mycoplasma agalactiae: Key Elements Involved in Horizontal Dissemination and Influence of Coresident ICEs
Source: mBio. 2018 Jul 3;9(4):e00873-18. doi: 10.1128/mBio.00873-18 (PMC6030558; doi:10.1128/mBio.00873-18)
Supplement: TABLE S4 [file mbo004183966st4.docx]

**Table S4.** Proteomic analysis of PG2 ICEA mutants. ^a^

| **Trypsin-digested products (AAs)** | **PG2^T^[ICEA *ncr19/E*::mTn]^G^23** | **PG2^T^[ICEA *cdsE*::mTn]^G^25** | **PG2^T^[ICEA *cds14*::mTn]^G^28** | **PG2^T^[ICEA *cdsF*::mTn]^G^35** | **PG2^T^[ICEA cds*H*::mTn]^G^43** |
| --- | --- | --- | --- | --- | --- |
| CDS1 (264) | - | - | - | - | - |
| CDSA (337) | - | - | - | - | - |
| CDS12 (134) | - | - | - | - | - |
| CDS11 (221) | - | - | - | - | - |
| CDS B (150) | - | - | - | - | - |
| CDSC (186) | 3 (21.0) ^b^ | 3 (21.0) | - | 1 (6.5) | 1 (6.5) |
| CDSD (217) | 2 (6.5) | 3 (12.9) | - | 2 (9.2) | 1 (6.5) |
| CDS5 (670) | - | - | - | - | - |
| CDS7 (326) | 2 (8.9) | 2 (8.9) | - | 2 (8.9) | - |
| CDS13 (84) | - | - | - | - | - |
| CDS15 (121) | 1 (18.2) | - | - | - | - |
| CDS16 (357) | - | - | - | - | - |
| CDS27 (86) | - | - | - | - | - |
| CDS17 (928) | * ^c^ | - | - | - | - |
| CDS19 (1517) | 2 (1.3) | - | - | 2 (1.8) | - |
| CDSE (150) | 4 (28.7) | - | 2 (21.3) | 4 (28.7) | 4 (36.7) |
| CDS14 (524) | 18 (40.8) | 18 (40.1) | - | 16 (40.8) | 11 (29.2) |
| CDSF (420) | 7 (20.0) | 4 (11.9) | 8 (21.9) | - | 3 (9.3) |
| CDS30 (117) | 8 (65.8) | 8 (65.8) | 8 (58.1) | 9 (71.8) | 9 (58.1) |
| CDSG (279) | - | - | - | - | - |
| CDSH (223) | - | - | - | - | - |
| CDS36 (64) | - | - | - | - | - |
| CDS22 (378) | - | - | - | - | - |

^a^ Cut-off values for proteins >250 AA were: unique peptide number >1 and total scan number >1; cut-off values for proteins ≤250 AA were: unique peptide number ≥1 and total scan number ≥2; ^b^ Unique peptides (coverage); ^c^ a single CDS 17 peptide was sporadically detected in other PG2 ICEA cells harboring a mTn inserted in *ncr19/E* (data not shown).
